# Statement of Retraction: Optimization of trypsin extraction technology of Allium cepa L. polysaccharide by response surface methodology and the antitumor effects through immunomodulation

**DOI:** 10.1080/21655979.2022.2145698

**Published:** 2022-11-22

We, the Editors and Publisher of the journal *Bioengineered*, have retracted the following article:

Yu-Bin Ji & Fu-Ling Wang. Optimization of trypsin extraction technology of Allium cepa L. polysaccharide by response surface methodology and the antitumor effects through immunomodulation. Bioengineered; 2021. 12(1):382-391. doi: 10.1080/21655979.2020.1870320.

Since publication, concerns have been raised about the integrity of the data in the article. When approached for an explanation, the authors have been unable to address the concerns raised and have not provided their original data in the appropriate format. As verifying the validity of published work is core to the integrity of the scholarly record, we are therefore retracting the article. The corresponding author listed in this publication has been informed.

We have been informed in our decision-making by our policy on publishing ethics and integrity and the COPE guidelines on retractions.

The retracted article will remain online to maintain the scholarly record, but it will be digitally watermarked on each page as ‘Retracted’.

